# Neuroinflammation by Cytotoxic T-Lymphocytes Impairs Retrograde Axonal Transport in an Oligodendrocyte Mutant Mouse

**DOI:** 10.1371/journal.pone.0042554

**Published:** 2012-08-08

**Authors:** Chi Wang Ip, Antje Kroner, Janos Groh, Marianne Huber, Dennis Klein, Irene Spahn, Ricarda Diem, Sarah K. Williams, Klaus-Armin Nave, Julia M. Edgar, Rudolf Martini

**Affiliations:** 1 Department of Neurology, Section of Developmental Neurobiology, University of Würzburg, Würzburg, Germany; 2 Department of Neuro-oncology, University Hospital, Heidelberg, Germany; 3 Department of Neurogenetics, Max Planck Institute of Experimental Medicine, Goettingen, Germany; 4 Applied Neurobiology Group, Institute of Infection, Immunity and Inflammation, College of Medical, Veterinary and Life Sciences, University of Glasgow, Glasgow, Scotland, United Kingdom; Johannes Gutenberg University of Mainz, Germany

## Abstract

Mice overexpressing proteolipid protein (PLP) develop a leukodystrophy-like disease involving cytotoxic, CD8+ T-lymphocytes. Here we show that these cytotoxic T-lymphocytes perturb retrograde axonal transport. Using fluorogold stereotactically injected into the colliculus superior, we found that PLP overexpression in oligodendrocytes led to significantly reduced retrograde axonal transport in retina ganglion cell axons. We also observed an accumulation of mitochondria in the juxtaparanodal axonal swellings, indicative for a disturbed axonal transport. PLP overexpression in the absence of T-lymphocytes rescued retrograde axonal transport defects and abolished axonal swellings. Bone marrow transfer from wildtype mice, but not from perforin- or granzyme B-deficient mutants, into lymphocyte-deficient PLP mutant mice led again to impaired axonal transport and the formation of axonal swellings, which are predominantly located at the juxtaparanodal region. This demonstrates that the adaptive immune system, including cytotoxic T-lymphocytes which release perforin and granzyme B, are necessary to perturb axonal integrity in the PLP-transgenic disease model. Based on our observations, so far not attended molecular and cellular players belonging to the immune system should be considered to understand pathogenesis in inherited myelin disorders with progressive axonal damage.

## Introduction

An important consequence of many myelin disorders is the degeneration of axons. Although it is well established that myelin and glial perturbation often leads to axon damage, the mechanisms involved are not yet entirely understood. Early transplantation studies performed in the peripheral nervous system using nerve segments from *trembler* mice unequivocally demonstrated that glial cells can locally influence axonal properties including axonal transport [1]. Other studies in the central nervous system on mice deficient in PLP or 2′, 3**′** -cyclic nucleotide 3′-phosphodiesterase also showed that mutant myelinating cells impair retrograde axonal transport [2] or cause features indicative of defective axonal transport [3], revealing a tight link beween the molecular integrity of myelinating glial cells and maintenance of axons [4], [5].

Importantly, most studies focussing on glia-related axon transport impairment were considering a two-cell scenario, comprising an abnormal myelinating glial cell and the axon directly affected by glial abnormalities by mainly unknown mechanisms. Using mice overexpressing PLP and serving as a model for X-linked spastic paraplegia type-2 [6], [7] our laboratory recently identified cytotoxic T-lymphocytes as mediators of primarily gliogenic neural damage [8], [9], [10], [11]. However, it was not investigated whether the low-grade inflammation also affected axonal transport.

In the present study, we specifically investigated the impact of neuroinflammation on retrograde axonal transport, a reliable parameter for examining axonal integrity [2], [12], [13], [14]. Of note, impaired axonal transport is also a pathological feature of various adult onset neurodegenerative diseases like Alzheimer’s disease, Huntington’s disease, motor neuron diseases or Parkinson’s disease [15], [16], [17], [18] and, interestingly, these disorders have often been found as being associated with inflammation of pathogenic relevance [19], [20].

We found that in PLP overexpressing mutants, the presence of functional cytotoxic T-cells is mandatory for glia-induced impairment of retrograde axonal transport and that this pathogenic effect is mediated by perforin and granzyme B. This finding substantially extends our knowledge about the pathomechanisms underlying primarily gliogenic axon perturbation.

## Results

### Compounds of the Adaptive Immune System Reduce the Efficacy of Retrograde Transport in PLP-tg Mice

**Figure 1:**
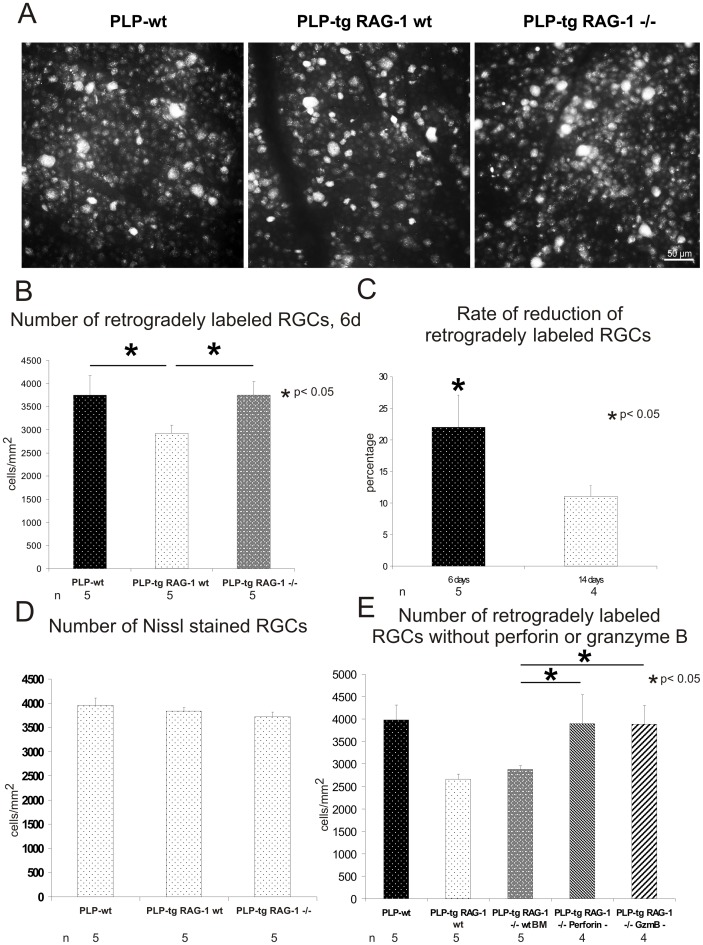
Retrograde transport is impaired in PLP-tg mutants, but reconstituted in the absence of the adaptive immune system and its cytotoxic molecules perforin and granzyme B. A) Retina flat mounts (middle region) from 10 months-old wild type (PLP-wt), PLP-tg (PLP-tg RAG-1 wt) and immune-deficient PLP-tg (PLP-tg RAG-1−/−) mice after 6 days of retrograde axonal transport of fluorogold. Different cell sizes and fluorogold intensity of labeled RGCs are visible. Note that in immune-competent PLP-tg mice, less retrogradely labeled RGCs are detectable than in PLP-wt, where nearly all RGCs are retrogradely labeled. In immune-deficient PLP-tg mice, the number of labeled RGCs is comparable to that of PLP-wt mice. B) Quantification of retrogradely labeled RGCs of PLP-wt (n = 5), PLP-tg RAG-1 wt (n = 5) and PLP-tg RAG-1−/− (n = 5) mice. In comparison to PLP-wt mice, less RGCs were labeled in PLP-tg mice. In PLP-tg RAG-1−/− mice, a similar number of RGCs was labeled as in PLP-wt mice. C) Relative reduction of retrogradely-labeled RGCs of PLP-tg (n = 5) in comparison to PLP-wt (n = 4) mice, dependent on the time period for retrograde axonal transport. The extention of the time period for retrograde transport of fluorogold from 6 to 14 days leads to a drop of reduced retrograde labeling of RGCs, indicating that retrograde axonal transport, rather than the continuity of the axons, is impaired. *significant reduction of labeled RGCs in comparison to wt mice (p<0.05). D) Quantification of Nissl-stained RGCs of PLP-wt (n = 5), PLP-tg RAG-1 wt (n = 5) and PLP-tg RAG-1−/− (n = 5) mice. Note that the number of Nissl-stained RGCs is not reduced in the PLP-tg mutants, as opposed to retrogradely labeled RGCs (Figure 1B). E) Quantification of retrogradely labeled RGCs (6 days period for retrograde axonal transport) of PLP-wt (n = 5), PLP-tg RAG-1 wt (n = 5) and PLP-tg RAG-1−/− mice reconstituted with bone marrow (BM) from either wild type mice (PLP-tg RAG-1−/− wt BM, n = 5), perforin- (PLP-tg RAG-1−/− Perforin -, n = 4) or granzyme-B-deficient mice (PLP-tg RAG-1−/− GzmB -, n = 4). Note that lack of either cytotoxic perforin or granzyme B leads to a reconstitution of retrograde axonal transport to PLP-wt level.

To investigate whether axonal transport is impaired in PLP overexpressing (PLP-tg) mice and, eventually, whether the immune system is involved in this potential perturbation, we first analyzed the axonal transport by retrograde labeling of retina ganglion cells (RGCs) after injection of fluorogold into the colliculus superior. 6 days after tracer injection, we counted 22% less labeled RGCs in the PLP-tg mutants compared to wt mice (p<0.05) (Figure 1A, B). Interestingly, when the time period for retrograde axonal transport was extended from 6 to 14 days, the reduction of labeled RGCs in PLP-tg mutants dropped to 11% and was no longer statistically significant (Figure 1C). This amelioration by an extended time period indicates that in the mutants, the efficacy of retrograde axonal transport was substantially reduced, and that axonal transection cannot be the major reason for the reduced number of labeled RGCs. Additionally, we counted the number of RGCs in flat mount preparations using histochemical (Nissl) staining. In both PLP-wt mice and PLP-tg mice, a comparable number of RGCs was detectable (Figure 1D), indicating that the oligodendrogliopathy did not lead to considerable neuronal cell death. Thus, in the PLP-tg mice, the efficacy of retrograde axonal transport was substantially reduced, but axonal transection was minor.

Next, we examined the efficacy of retrograde axonal transport in PLP-tg RAG-1−/− mice that lack functional T-and B-lymphocytes (but not NK cells) due to a null mutation in the recombination activating gene (RAG)-1 [8]. In the tracing experiments with a transport time of 6 days, we found more retrogradely labeled RGCs in the PLP-tg RAG-1−/− mice than in immune-competent PLP-tg mutants (PLP-tg RAG-1 wt), with values similar to those obtained by PLP-wt mice (Figure 1A, B).

Then we investigated whether perforin and granzyme B, the typical cytotoxic mediators of CD8-positive T-lymphocytes, also disturb retrograde axonal transport. We created chimeric mice by bone marrow transplantation from either perforin- or granzyme B-deficient mutants into PLP-tg RAG-1-deficient mice. We found that in PLP-tg mutants with T-lymphocytes lacking perforin or granzyme B, the number of retrogradely labeled RGCs was at the level of wt mice (Figure 1E), showing that perforin and granzyme B are important mediators of perturbation of retrograde axonal transport in the myelin mutants. Of note, the T-lymphocytes lacking perforin or granzyme B invaded the mutant CNS to a similar degree as in genuine PLP-tg mice (not shown). Control transplantation experiments with wt bone marrow led to a reduction of retrogradely labeled RGCs comparable to PLP-tg RAG-1 wt mice (Figure 1E).

### Juxtaparanodes are Perturbed in Immunocompetent PLP-tg Mice

**Figure 2:**
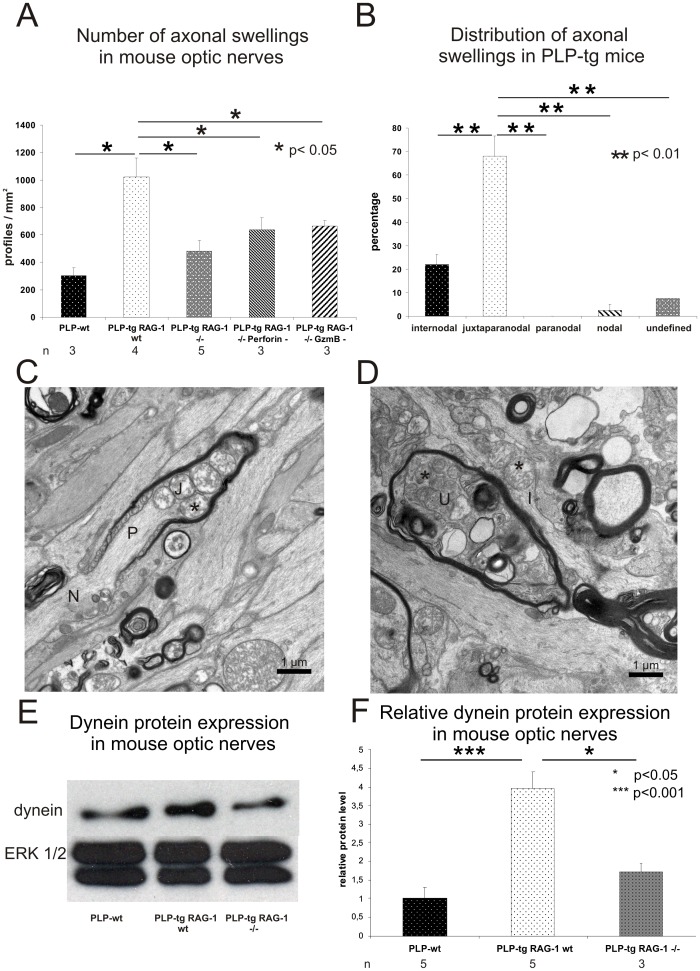
The occurrence of axonal swellings depend on lymphocytes, cytotoxic perforin or granzyme B and are located at juxtaparanodal aspects. A) Number of axonal swellings (Bielschowsky’s silver impregnation, optic nerve) in PLP-wt (n = 3), PLP-tg RAG-1 wt (n = 4), PLP-tg RAG-1−/− (n = 5) mice and in PLP-tg mice devoid of cytotoxic perforin (n = 3) or granzyme B (n = 3). RAG-1 deficiency and lack of cytotoxic perforin or granzyme B in PLP-tg mice leads to a significant reduction of axonal swellings (p<0.05, one-sided student’s t-test). B) Quantification of axonal swellings in 3 PLP-tg RAG-1 wt mice by electron microscopy displays that the majority of these abnormalities are located at the juxtaparanodal region while only few abnormally organized structures are located at other domains of the myelinated fiber. Note that the paranode is not abnormally organized. C, D) Electron microscopy of PLP-tg mouse optic nerves identifies abnormal juxtaparanodal and internodal profiles, containing mitochondria (asterisks), often with an appearance reminiscent of degeneration. An axonal swelling in an undefined region is marked by an “U”. N: Node of Ranvier; P: Paranode; J: Juxtaparanode; I: Internode. E, F) Western blot analysis of optic nerves of PLP-wt, PLP-tg RAG-1 wt and PLP-tg RAG-1−/− mice. E) In this example with one individual for each genotype, dynein levels are elevated in PLP-tg RAG-1 wt mice compared to PLP-wt. RAG-1 deficiency leads to dynein levels in PLP-tg mice comparable to those found in PLP-wt mice. ERK1/2 was used as loading control. F) Quantitative Western blot analysis using densitometry and 3–5 individuals per genotype confirm the elevated dynein levels in PLP-tg RAG-1 wt mice compared to PLP-wt. RAG-1 deficiency leads to dynein levels in PLP-tg mice comparable to those found in PLP-wt mice.

Using Bielschowsky’s silver impregnation, axon enlargements/swellings were identified as correlates of axonal perturbation in PLP-tg mice (Figure 2A). Furthermore, RAG-1-, perforin- and granzyme B-deficiency caused a significant reduction of such abnormalities compared to immune-competent PLP-tg mice (Figure 2A).

We now further characterized the axonal abnormalities within optic nerves of 3 PLP-tg mice by electron microscopy to determine their relative positions along the myelin sheath. The majority of axonal enlargements was located at the juxtaparanodal region distal to the node of Ranvier with regard of the retina ganglion cell bodies (Figure 2B, C). Typically, there was a substantial accumulation of mitochondria. Sometimes, the mitochondria appeared though as if they would “plug” the axon constriction at the transition between the paranode and the juxtaparanode (Figure 2C). The mitochondria, furthermore, often showed morphological alterations, ranging from almost normal appearance to contorted structures reminiscent of mitochondria fused to lysosomes (Figure 2C, D).

### Dynein Protein Level is Increased in PLP-tg Mice

Dynein is the major motorprotein mediating the retrograde axonal transport. Because of the impaired retrograde axonal transport in PLP-tg mice, we therefore analyzed the protein levels of dynein in triton-soluble extracts of PLP-tg mouse optic nerves. Western blot analysis showed an increase of dynein protein level in PLP-tg mouse optic nerve compared to extracts from optic nerves of PLP-wt mice. RAG-1 deficiency led to dynein levels comparable to those seen in PLP-wt mice in PLP-tg mice (Figure 2E, F).

### CD8+ T-lymphocytes Contact Juxtaparanodal Regions where they Acquire an Elongated Shape

**Figure 3:**
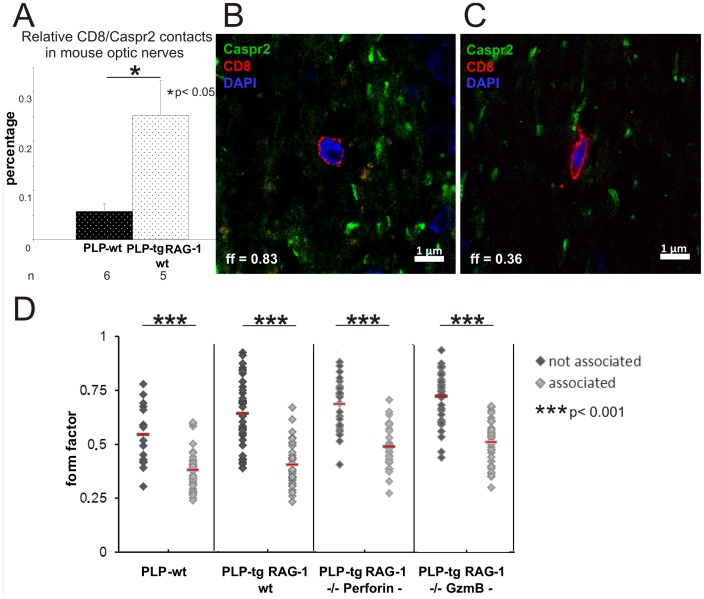
CD8+ T-lymphocytes contact juxtaparanodes where they acquire a spindle-shaped form. A) Relative number of CD8/Caspr2 contacts in PLP-wt (n = 6) and PLP-tg (n = 5) mouse optic nerves. In PLP-tg mice, significantly more CD8+ T-lymphocyte-to-juxtaparanode contacts are visible than in the PLP-wt mice. B, C) Double immunofluorescence in mouse optic nerve for CD8+ T-lymphocytes (red) and Caspr2+ juxtaparanodal regions (green). Cell nuclei are stained blue with DAPI. T-lymphocytes devoid of cell contact to Caspr2+ structures are preferentially rounded (B) while T-lymphocytes attached to Caspr2+ structures are spindle shaped (C). D) Dot-plot showing form factor values of individual T-lymphocytes in PLP-wt, PLP-tg and PLP-tg mice without perforin or granzyme B. At least 16 Caspr2-associated and not-associated T-lymphocytes were investigated in each genotype (n = 2–3). Independent of the genotype, CD8+ T-lymphocytes display a low form factor representing a spindle-like shape when associated with the juxtaparanodal region.

To further investigate whether there is a spatial relationship between CD8+ T-lymphocytes and juxtaparanodal regions, we performed double immunofluorescence using antibodies against CD8 and Caspr2. Indeed, we often observed a close association between CD8+ T-lymphocytes and Caspr2+ profiles in all genotypes investigated. In PLP-tg mice, we found approximately 4-fold more CD8+ T-lymphocyte-to-juxtaparanode contacts in relation to the number of juxtaparanodes than in PLP-wt mice (0.25% vs 0.06%, p<0.05) (Figure 3A). Additionally, confocal microscopy revealed that in PLP-tg mice, 41.3% ±11.6% of all CD8+ T-lymphocytes in the optic nerve sections are attached to the juxtaparanodes. Of note, CD8+ T-lymphocytes remote to Caspr2+ profiles were mostly rounded, whereas CD8+ T-lymphocytes at juxtaparanodal regions preferentially displayed an elongated shape (Figure 3B, C). As a quantitative measure, this was reflected by a higher form factor (representing a more rounded shape, see Materials and Methods) in the former group and by a lower form factor of T-lymphocytes associated with Caspr2+ profiles (Figure 3B–D). Interestingly, the elongated, attaching T-lymphocytes often displayed protrusions from one end of the cell body that contacted the juxtaparanode (Figure 3C).

## Discussion

In the present study, we could unequivocally demonstrate that lymphocytes mediate axonal perturbation in PLP overexpressing mutant mice. As typical for cytotoxic T-lymphocytes this impairment was most likely mediated by perforin and granzyme B. These findings do not only identify some elements of the basic mechanisms of axon perturbation in PLP mutants, but also demonstrate that at least in the present mutant, glial perturbation alone is not sufficient for robust axon impairment, but needs the involvement of a “third“ cellular component, the immune cell. This is clearly reflected by normal, wild-type-like efficacy of axonal transport in the presence of the glial mutation, but in the absence of lymphocytes or when lymphocytic effector cells are molecularly impaired.

Our study strongly suggests that reduced retrograde labeling in the optic system is mediated by impaired retrograde axonal transport *per se*, rather then by complete axonal transection which would lead to a reduced number of labeled retina ganglion cell bodies as well. Of note, in a related mutant, simultaneous injections of axonal tracers into the superior colliculus (retrograde transport) and the retina (anterograde transport) identified double-labeled axonal bulbs reflecting axonal continuity in the presence of axonal abnormalities and impaired retrograde axonal transport [21]. For the present study, there are at least two arguments strongly favouring the continuity of the vast majority of retina ganglion cell axons. First, extention of the time period for retrograde axonal transport leads to a substantial increase of retrogradely labeled neuronal cell bodies in the PLP mutants. This shows that, in the PLP mutants, axons transport the tracer with a reduced efficacy and need extended time periods to generate detectable labeling levels in some cell bodies. Reciprocally, if reduced numbers of labeled retinal ganglion cell bodies would have been caused predominantly by axonal transection, it is unlikely that extended time periods for retrograde axonal transport would have elevated the number of labeled cell bodies in the retina. Second, as a more indirect argument, retinal ganglion neurons are highly susceptible to cell death when axons are completely transected, either mechanically [22], [23] or by neuroinflammation [12]. In our study, we could neither find a significant reduction of retina ganglion cells nor pyknotic cell nuclei by histological stainings, suggesting that axonal continuity is mostly preserved in the PLP mutants. Thus, in the PLP mutants, axons appear morphologically altered [8], but not entirely transected. This has important implications for therapies aimed at rescuing injured axons, because it demonstrates the potential reversibility of such axonal changes [24].

To further elucidate the nature of disturbed retrograde axonal transport in PLP overexpressing mice we investigated the motor protein for retrograde transport in optic nerve lysates. A significant increase of dynein protein level was detected in PLP-tg mouse optic nerves compared to PLP-wt mice. Similar results with elevated dynein levels have been observed in PLP-null mice that also show disturbed axonal transport [2]. It has been suggested that raised dynein protein levels might reflect an accumulation of dynein-linked retrogradely moving organelles [2]. This is in line with our finding that juxtaparanodal axonal swellings with morphologically-altered mitochondria were located predominantly on the distal side of the node of Ranvier (in regard to the retina). Accumulation of mitochondria or other cell organelles in axonal abnormalities are often a correlate for disturbed axonal transport [2], [25], [26], [27] thus suggesting a link between the impaired retrograde axonal transport and the formation of the juxtaparanodal swellings and increase in dynein protein levels. These features were nearly restored in the absence of RAG-1 in PLP-tg mice suggesting a central role of adaptive immune cells in axonopathic changes in the respective mutants.

Since in our study, juxtaparanodes appeared to be most susceptible for changes mediated by the cytotoxic T-lymphocytes, we investigated the possible association of these structures. Indeed, T-lymphocytes in direct vicinity to juxtaparanodes were detectable and, most interestingly, preferentially displayed a spindle-like shape at this location. We do presently not know the significance of this constant observation, but it is worthwhile to speculate that increased adhesion to a possible target structure might lead to the maximal cell contact extension between lymphocytes and the juxtaparanode resulting in the elongated shape of the lymphocytes. Interestingly, this phenomenon has been observed also in perforin- and granzyme B-deficient myelin mutants suggesting that this cell-cell interaction is independent of the cytotoxic features of the lymphocytes. Whether typical axo-glial molecules, which can even serve as antigens in multiple sclerosis [28], are functionally involved in this interaction, remains to be resolved.

Our basic finding that cytotoxic lymphocytes with their respective cytotoxic agents are essential for impaired retrograde axonal transport is reminiscent of the pathomechanisms described in Theiler’s virus-related model of demyelination [29], [30], [31], [32]. However, in contrast to the Theiler’s virus model, in PLP transgenic mice MHC class I immunoreactivity is only detectable on the mutant oligodendrocytes [8]. This might have substantial consequences for the respective pathomechanisms. For the Theiler’s virus model, it is assumed that the virus-mediated demyelination exposing the MHC class I-positive axolemma is an important prerequisite for the formation of an immunological synapse between lymphocytes and MHC class I-positive axons and, thus eventually, for the immune attack by the cytotoxic T-cells [29], [30], [31], [32]. In the PLP mutant, in which MHC I-restricted T-cell receptors play a crucial role for neural damage [10], cytotoxic T-cells might only be able to attack the MHC class I-positive oligodendrocytes, since the molecule is not detectable on axons [8]. Paradoxically, in the PLP mutants, myelin often remains intact over wide stretches of the internodes whereas the axons show shrinkage or swelling causing periaxonal vacuoles [8] and axonal enlargements [10], respectively. This scenario resembles pathological features seen in MOG-EAE, where the immunological attack against the target cell, the oligodendrocyte, is transmitted to the axon without major local damage to myelin [24], [33]. How the glial-related attack is transmitted to the axonal partner and whether the spindle-shaped lymphocytes (see above) directly attaching to the juxtaparanodes are the cytotoxic mediators is presently not clear.

The primary defect of PLP transgenic mice clearly resides in mutant oligodendrocytes. Axonal pathology, on the other hand, is strongly reduced on the RAG-1−/− background, but unlike the absence of lymphocytes not completely abolished [8]. Thus, secondary axonal swellings and periaxonal vacuoles in a primary glial disease can – at a low level - principally exist also independent of invading cytotoxic T-lymphocytes, which therefore emerge as substantial ‘amplifiers’ of diseases. The cytotoxic attack by perforin and granzyme B might alter the subcellular organisation of oligodendrocytes causing impairment of normal diffusion and transport processes within cytosolic channels of myelin, hypothesized to play an important role in the oligodendroglial support of axon function [4].

Alternatively, it is possible that a “spillover” of perforin and granzyme(s) from invading T-cells is a collateral damage and bystander effect that directly perturbs axon functions, as hypothesized for experimental *ex vivo* models [34], [35], [36]. In this context, it is striking that released granzyme B can damage neurons via interaction with the neuronal mannose-6-phosphate-receptor [37], [38] which is not only located on neuronal somata and dendrites, but also on axons [39]. In this model, the antigen-specific cytotoxic attack to glial cells would release perforin/granzyme B to diffuse along the myelinated fiber, eventually binding to axonal mannose-6-phosphate receptor at the nodes of Ranvier. This could lead to endocytosis of the granzyme B-mannose-6-phosphate receptor-complex and release of granzyme B into the axoplasm by a perforin-dependent process [38]. Once granzyme B has been transfered into the cytoplasm, it could promote reorganization of microtubules [40] or mitochondrial damage [38] leading to impaired retrograde axonal transport. Why, however, axonal changes are predominantly seen at juxtaparanodes rather than at the node proper, can presently not be explained by this model.

Irrespective of the exact pathomechanism, blocking inflammation in this model might be beneficial for the preservation of axon transport and for the maintenance of the integrity of critical axonal compartments, such as the juxtaparanode with its pivotal physiological functions [41]. Of note, many neurological disorders are associated with impaired retrograde axonal transport and impaired axonal transport itself may also have a pathogenic impact so that improvement of axonal transport might be a therapeutic target to ameliorate disease [15], [16], [17], [18], [42], [43]. Based on our findings, one way to improve axonal transport might be to attenuate inflammation. Our study has therefore clear implications also for the development of treatment strategies in the group of inherited myelin disorders and possibly other neurodegenerative disorders that have an inflammatory component and are marked by progressive impairment of axon function.

## Materials and Methods

### Ethics

Animal experiments were approved by the Regierung von Unterfranken Wuerzburg.

### Animals

10 months old heterozygous PLP overexpressing mice (PLP-tg; PLP-tg RAG-1 wt) of the line 66 and PLP-tg RAG-1−/− mutants were examined. Perforin-deficient mice were kindly provided by T. Hünig (Wuerzburg). Granzyme B−/− mice were obtained from the Jackson Laboratory (Bar Harbor, ME). All investigated mice were on a C57BL/6 background and were kept in our animal facility under barrier conditions.

Bone marrow transplantation from wt, perforin- or granzyme B-deficient donor mice into 8 weeks old PLP-tg RAG-1−/− mutants (hosts) and control of successful transplantation were performed as described before [10]. Hosts were sacrificed after 8 months of survival.

### Retrograde Labeling of Retina Ganglion Cells (RGCs)

Mice were anesthetized (intraperitoneally, Ketamin/Rompun) and placed into a stereotactic frame. RGCs were retrogradely labeled with 1.5 µl fluorogold (4% in 0.9% saline; Fluorochrome Inc., Englewood, Colorado). Injections were given into both colliculi superiores using stereotactic coordinates (bregma 3.6 mm caudally, 0.6 mm laterally, and 1.75 mm ventrally). After 6 or 14 days, mice were perfused with 0.9% saline and retinae were prepared as whole mounts (four retinal leafs) and immersion-fixed in 4% PFA/PBS (40′). For quantification of RGCs, 12 digital images were acquired per retina (inner, middle, outer region of the flattened retina), corresponding to locations at 1/6, 3/6 and 5/6 of the retinal radius. All retrogradely labeled RGCs were quantified independent of size.

### CD8/Caspr2 Double-Immunohistochemistry

Freshly dissected mouse optic nerves were snap frozen in liquid nitrogen-cooled isopentane. 10 µm thick longitudinal kryosections were cut and immersion fixed in 2% PFA/PBS for 10 minutes at room temperature (RT). After washing in PBS, sections were incubated in 4% FCS/4% NGS in 0.3% Triton-X-100/PBS for 1 h at RT. Afterwards primary antibodies rat-anti-mouse-CD8 (Chemicon, Temecula, CA) and rabbit-anti-mouse-Caspr2 as a marker for juxtaparanodes (AB5886; Millipore, Billerica, USA) diluted in blocking solution were applied for two hours at RT, followed by incubation with fluorescence-labeled secondary antibodies (goat-anti-rat-Cy3, Dianova, Hamburg, Germany; goat-anti-rabbit-Alexa Flour 488, A11008, Invitrogen, Karlsruhe, Germany). In order to mark cellular nuclei the sections were incubated with DAPI (Sigma-Aldrich, Munich, Germany; 1∶500000) and then embedded in Aqua-Poly/Mount. DAPI positive CD8+ T-lymphocytes and Caspr2+ profiles were counted per mm^2^ in myelinated optic nerve with the confocal microscope FluoView FV1000 (Olympus, Hamburg, Germany). Every CD8+ T-cell was completely scanned with 1 µm stacks. For the analysis of association rate, the percentage of CD8+ T-lymphocytes attatched to juxtaparanodal regions was quantified in relation to the number of Caspr2+ profiles. Of each cell the stack with the largest surface area was measured with ImageJ 1.45 (National Institutes of Health, Bethesda, MD) for area and perimeter. According to the formula for the form factor ( = 4 π× cell area/perimeter^2^; ref. [44]) values were obtained and compared between different genotypes. All sections were analysed by the investigator not being aware of the genotype.

### Bielschowsky’s Silver Impregnation

10 µm optic nerve sections were incubated in 20% lead nitrate for 30 minutes at room temperature, followed by incubation in 4% formaldehyde (10 sec). Sections were then impregnated in lead nitrate/ammonium hydroxide solution for 30 seconds. After washing in distilled water, sections were incubated in 5% sodiumthiosulfatesolution.

### Electron Microscopy

Optic nerves of transcardially perfused mice (4% PFA/2% GA) were osmificated and embedded in Spurr’s medium. 70 nm ultrathin sections were counterstained with lead citrate and examined using Leo 906E electron microscope (Zeiss, Oberkochen, Germany), equipped with a ProScan CCD camera (Lagerlechfeld, Germany).

### Western Blot Analysis

Optic nerves were quickly dissected, snap frozen in liquid nitrogen and sonicated (Sonoplus HD60, Bandelin electronic, Berlin, Germany) in 100 µl RIPA lysis buffer (25 mM Tris-HCl, pH 8, 10 mM Hepes, 150 mM NaCl, 145 mM KCl, 5 mM MgCl2, 2 mM EDTA, 0.1% SDS, 1% NP-40, 10% Glycerol with protease inhibitors) per 10 mg tissue. In addition, triton soluble (membrane protein enriched) and triton insoluble fractions of optic nerve lysates were prepared according to previously published protocols [2]. Protein concentration was determined by Lowry assay (Sigma-Aldrich) and equal amounts of proteins were resolved by SDS-PAGE, transferred to nitrocellulose membranes and visualized using Ponceau S (Roth, Karlsruhe, Germany). Membranes were blocked with skim milk and probed with anti-dynein antibody solution (intermediate chain) overnight at 4°C (Serotec, Oxford, UK). Incubation with HRP-conjugated secondary antibodies was performed for 1 h at room temperature and detection of the immune reaction was achieved by use of ECL reagent and ECL hyperfilm (GE Healthcare Bio-Sciences AB, Uppsala, Sweden). The resulting signals for motor proteins were normalized to the amount of the corresponding signal of the loading control following densitometric quantification using ImageJ software (National Institutes of Health, Bethesda, MD).

### Statistical Analysis

Statistical analysis was performed by using the unpaired two-tailed student’s t-test for comparison of quantified profiles. For analysis of axonal swellings a one-tailed student’s t-test was used. Relative reduction of retrogradely labeled RGCs was determined by calculating the percentage of RGCs in PLP-tg RAG-1 wt mouse retinae in relation to the number of RGCs of corresponding PLP-wt mice.
